# Different pieces of the same puzzle: a multifaceted perspective on the complex biological basis of Parkinson’s disease

**DOI:** 10.1038/s41531-023-00535-8

**Published:** 2023-07-13

**Authors:** Amica C. Müller-Nedebock, Marieke C. J. Dekker, Matthew J. Farrer, Nobutaka Hattori, Shen-Yang Lim, George D. Mellick, Irena Rektorová, Mohamed Salama, Artur F. S. Schuh, A. Jon Stoessl, Carolyn M. Sue, Ai Huey Tan, Rene L. Vidal, Christine Klein, Soraya Bardien

**Affiliations:** 1 Division of Molecular Biology and Human Genetics, Department of Biomedical Sciences, Faculty of Medicine and Health Sciences, Stellenbosch University, Cape Town, South Africa; 2 South African Medical Research Council/Stellenbosch University Genomics of Brain Disorders Research Unit, Stellenbosch University, Cape Town, South Africa; 3 Department of Internal Medicine, Kilimanjaro Christian Medical Centre, Moshi, Tanzania; 4 Norman Fixel Institute for Neurological Diseases, McKnight Brain Institute, University of Florida, Gainesville, FL USA; 5 Research Institute of Disease of Old Age, Graduate School of Medicine, Juntendo University, 2-1-1 Hongo, Bunkyo-ku, Tokyo 113-8421 Japan; 6 Department of Neurology, Juntendo University School of Medicine, 2-1-1 Hongo, Bunkyo-ku, Tokyo 113-8421 Japan; 7 Neurodegenerative Disorders Collaborative Laboratory, RIKEN Center for Brain Science, 2-1 Hirosawa, Wako, Saitama 351-0106 Japan; 8 Division of Neurology, Department of Medicine, Faculty of Medicine, University of Malaya, Kuala Lumpur, Malaysia; 9 The Mah Pooi Soo & Tan Chin Nam Centre for Parkinson’s & Related Disorders, Faculty of Medicine, University of Malaya, Kuala Lumpur, Malaysia; 10 Griffith Institute of Drug Discovery (GRIDD), Griffith University, Brisbane, QLD Australia; 11 First Department of Neurology and International Clinical Research Center, St. Anne’s University Hospital and Faculty of Medicine, Masaryk University, Brno, Czech Republic; 12 Applied Neuroscience Research Group, CEITEC, Masaryk University, Brno, Czech Republic; 13 Institute of Global Health and Human Ecology (I-GHHE), The American University in Cairo (AUC), New Cairo, 11835 Egypt; 14 Faculty of Medicine, Mansoura University, Dakahleya, Egypt; 15 Atlantic Senior Fellow for Equity in Brain Health at the Global Brain Health Institute (GBHI), Trinity College Dublin (TCD), Dublin, Ireland; 16 Departamento de Farmacologia, Universidade Federal do Rio Grande do Sul, Porto Alegre, Brazil; 17 Serviço de Neurologia, Hospital de Clínicas de Porto Alegre, Porto Alegre, Brazil; 18 Pacific Parkinson’s Research Centre, Department of Medicine (Division of Neurology), Djavad Mowafaghian Centre for Brain Health, University of British Columbia, Vancouver, BC Canada; 19 Neuroscience Research Australia; Faculty of Medicine, University of New South Wales; Kinghorn Centre for Clinical Genomics, Garvan Institute of Medical Research, Darlinghurst; Department of Neurology, Prince of Wales Hospital, South Eastern Sydney Local Health District, Randwick, NSW Australia; 20 Instituto de Neurociencia Biomédica (BNI), Facultad de Medicina, Universidad de Chile, Santiago, Chile; 21 Centro FONDAP de Gerociencia, Salud Mental y Metabolismo (GERO), Santiago, Chile; 22 Centro de Biología Integrativa, Facultad de Ciencias, Universidad Mayor, Santiago, Chile; 23 Institute of Neurogenetics, University of Lübeck and University Hospital Schleswig-Holstein, Lübeck, Germany

**Keywords:** Parkinson's disease, Diseases

## Abstract

The biological basis of the neurodegenerative movement disorder, Parkinson’s disease (PD), is still unclear despite it being ‘discovered’ over 200 years ago in Western Medicine. Based on current PD knowledge, there are widely varying theories as to its pathobiology. The aim of this article was to explore some of these different theories by summarizing the viewpoints of laboratory and clinician scientists in the PD field, on the biological basis of the disease. To achieve this aim, we posed this question to thirteen “PD experts” from six continents (for global representation) and collated their personal opinions into this article. The views were varied, ranging from toxin exposure as a PD trigger, to *LRRK2* as a potential root cause, to toxic alpha-synuclein being the most important etiological contributor. Notably, there was also growing recognition that the definition of PD as a single disease should be reconsidered, perhaps each with its own unique pathobiology and treatment regimen.

## Introduction

Since the first description of Parkinson’s disease (PD) in Western Medicine over two centuries ago, significant progress has been made to better understand, diagnose and treat the disease^1^. Yet, despite this progress and years of research in the PD field, our understanding of its biological basis remains incomplete. Over the years, several different theories on the pathobiology of PD have emerged and evolved, with the general consensus being that the disease is complex with multiple factors (many still unknown) contributing to disease manifestation and progression.

The aim of this article was to explore some of these theories by asking PD clinician and laboratory scientists from around the globe to answer the question “*What do* *you* *consider to be the starting point and the process that leads to the development of PD, and why*?”. They could also comment on where future research efforts should be directed. Contributors were selected based on geographic spread, gender equity and diversity of their research interests. Overall, we had 12 contributions from six continents (Africa, Asia, Australia, Europe, North America, and South America). While authors sharing similar viewpoints were clustered together, their written and unedited views are presented here as a collection of their responses to the question.

## Pathogenic sequence variants in conjunction with mitochondrial dysfunction and alpha-synuclein accumulation

Genetic studies that led to the identification of now well-established PD genes, including *PRKN*, *PINK1*, *SNCA* and *LRRK2*^2^, have undoubtedly been key in elucidating the possible biological basis of the disease. One of our contributors highlights variants in these genes as an important contributing factor to the development of PD.

### Carolyn M. Sue, MD, PhD, Australia

As with most complex diseases, there are numerous pathological processes that can contribute to the development of a final common clinical phenotype such as PD. Contributions from each process vary amongst affected individuals in the context of the individual’s predisposition to develop the disorder. In PD, some individual’s genetic background often encodes their risk of developing PD and for other individuals, their vulnerability to the pathological processes that may be triggered by disease process precipitants (e.g., toxin exposure).

Pathogenic mutations in causative PD genes are some of the strongest predisposing risk factors^3^, with biallelic mutations in some genes (e.g., *PRKN*, *PINK1*) being, by and large, fully penetrant. Mutations in other PD genes (e.g., *GBA* and *LRRK2*) may increase an individual’s risk, and are not associated with full penetrance. The extent by which aging, environmental factors or changes in lifestyle behaviors can modify disease onset in these mutation carriers is the subject of current research endeavors^4^.

Wherever an individual lies along this genetic risk spectrum, the two most important cellular processes that contribute to PD pathogenesis are mitochondrial dysfunction and the accumulation of aggregated alpha-synuclein. Each patient with PD may have varying degrees of disruption to each of these cellular pathways that when perturbed, interact to result in a cascade of events that accelerates cellular dysfunction and ultimately leads to the accumulation of toxic protein species such as aggregated alpha-synuclein, and cell death. Strong disruptors of mitochondrial function such as MPTP (or more accurately, MPP+) or rotenone, can cause PD, regardless of genetic status. Milder causes of mitochondrial dysfunction, such as biological aging, lead to more modest reductions in bioenergetic function and a cascade of cellular events including the generation and release of excessive reactive oxygen species, abnormal intraorganellar trafficking, inefficient protein clearance, impaired lysosomal function and the accumulation of alpha-synuclein, all of which can contribute and feed forward to the neurodegenerative process in PD^5^.

By contrast, aggregations of alpha-synuclein may result from over production of endogenous alpha-synuclein (e.g., *SNCA* triplications) that can overwhelm otherwise efficient protein clearance pathways. Protein clearance may be impaired due to mutations in *GBA*, be overloaded with increased exposure to alpha-synuclein or fail as a consequence of reduced bioenergetic function associated with the aging process. Mitochondrial dysfunction can lead to impaired protein clearance and the subsequent accumulation of aggregated alpha-synuclein. Completing the vicious cycle, aggregated alpha-synuclein impairs mitochondrial and lysosomal function to accelerate the neurodegenerative process^6,7^.

## Leucine-rich repeat kinase 2 (*lrrk2*): a root cause?

As emphasized by one of our contributing authors below, *LRRK2*^8,9^, has been repeatedly linked to PD since its discovery in the early 2000s, with its encoded protein acting as a notorious multitasker that may contribute to PD’s biological basis in multiple ways.

### Matthew Farrer, PhD, USA

Increased LRRK2 kinase activity is the single most important causal factor as it is responsible for PD, whether sporadic^10^ or in families with a dominantly-inherited disease^8,9^. Importantly, *LRRK2*-parkinsonism is clinically indistinguishable from idiopathic late-onset PD^11^. Half of the genetically-identified *LRRK2* individuals with PD that come to autopsy manifest midbrain Lewy body disease^12^ and satisfy a definitive pathologic diagnosis of PD^13^. Although there have been no formal epidemiologic studies, and the incidence, prevalence and penetrance figures cited in the literature are mostly clinic-based^14^, *LRRK2* pathogenic substitutions appear to confer the highest individual genotypic/disease-penetrant and population-attributable risk of PD^15^. LRRK2 coding substitutions tend to be population specific; *LRRK2* p.R1441G in the Euskera Basques^16^, p.G2019S in North African Berbers^17,18^ and Ashkenazi Jews^19^, and p.G2385R in South Eastern Asia^20^, define “patches” of PD on a world map. The majority of these mutant alleles originated from one or few founders, their frequencies evolutionarily increased by recent positive selection^21^. What that selective allelic advantage is remains to be discovered. In support, LRRK2 is highly expressed in cells of myeloid-lineage in the peripheral immune system and in brain-resident microglia^22,23^, the *LRRK2* promoter contains interferon-gamma responsive elements^24^, and its level of expression is clearly responsive to inflammatory stimuli^25^. The locus has also been nominated in genome-wide association studies (GWAS) of idiopathic PD^26^, progressive supranuclear palsy^27^ and several chronic inflammatory disorders^28,29^. As the immune system mediates a host’s response to its environment, and PD has long been considered a multifactorial disorder^30^, it is attractive to hypothesize *successive periods of inflammation and LRRK2 activation may explain the penetrance of LRRK2-parkinsonism*, and such a mechanism may partially underlie the ontology and incidence of idiopathic PD that is steadily increasing. Hence, LRRK2 biology currently attracts much academic, philanthropic and pharmaceutical research interest and investment.

All *LRRK2* mutations that cause PD activate its kinase activity, directly or indirectly, the most evident being p.G2019S that keeps the hinge of kinase “activation segment” ajar^31^. Mutations in other Mendelian genes for dominantly-inherited PD, such as vacuolar protein sorting 35 p.D620N^32,33^, also result in constitutive LRRK2 kinase activation^34^. Hence, LRRK2 kinase inhibitors are now in human clinical trials given their promise to halt disease progression. Nevertheless, how LRRK2 kinase dysfunction manifests in the selective vulnerability of dopaminergic neurons^35^, and what inter- (dopaminergic and/or non-cell autonomous) and intra-cellular events underlie their demise is unknown. LRRK2 forms a large, dimeric protein scaffold with several lysosomal targeting motifs^36^ and protein-interaction domains^37^. It is intimately associated with endosomal-lysosomal processes, cytoskeletal and vesicular trafficking^38^, and can phosphorylate multiple substrates including a subset of Rab GTPases^39^. Therefore, LRRK2’s functions are consequently pleiotropic.

While there is now much data and synthesis, the question of what biology is necessary and sufficient for the ontology of PD remains unanswered, and how can that be elucidated? One approach to an integrated and holistic understanding is to use conditional cre-loxP animal models^40^ that recapitulate mutant gene dysfunction. As PD is multifactorial, and a function of the relationship between neurons, glia, the immune system and the environment, then models should have these components. It will also be necessary to compare findings between models of different mutant genes implicated in PD to identify their biological “intersection”. While the magnitude of locus associations nominated through GWAS are too small and too pleiotropic to drive comparable neuroscience^41^, implicating the proteins encoded in a mechanistic “intersection” would be useful and an important validation.

## Toxin exposure

Before the identification of genetic contributors such as *LRRK2*, PD was believed to be an archetypal “non-genetic” disease; a view that was supported by early observations that toxin exposure could cause a parkinsonism phenotype^42^. To date, toxin exposure is still considered an important but underappreciated (and understudied) contributor to PD that could give important insights into the disease’s pathobiology. This notion is also shared by two contributing authors whose opinions are provided below.

### Marieke Dekker, MD, PhD, Tanzania

Genetic susceptibility in combination with exogenous causes, including toxins, are thought to have a cumulative impact on the brain. This results in pathogenic protein accumulation, low-grade inflammation and loss of dopamine-producing neurons. Current disease hypotheses are largely based on research done in high-income countries. Let us focus on Sub-Saharan Africa (SSA), where within a single generation’s time, life expectancy has increased by 10 years to 62 years^43^, which has implications for age-related disorders such as PD. The mean age of onset of PD in a Tanzanian community-based study from 2008 was 69.4 years. However, a number of recent, mostly hospital-based, studies in SSA report a trend towards a higher prevalence and a possible lower age of PD onset of 55–60 years^44–47^ (unpublished data).

Africans are more genetically diverse among themselves than they are with respect to other ethnicities^48,49^. Presently known PD genes do not seem to play a significant role in Southern and Western African PD patients^47,49^. Whole genome screening in SSA PD patients is ongoing but very limited^47,50^. It is unclear whether the lower life expectancy in SSA underestimates genetic factors. It also begs the question of whether the magnitude of genetic risk factors for PD is different in SSA, with perhaps a larger role for inflammation or environmental toxins.

Inflammation in PD has been widely studied but regarded as consequence rather than a cause. No infectious cause has been found. There is epidemiological and in-vitro evidence of some causal link between pesticide exposure and PD, especially in rural populations^51^. Most of SSA inhabitants rely on subsistence farming, and pesticide use is omnipresent. The longer life expectancy and a young median age of the population make for a large cohort of individuals in rural SSA, who are at risk for potential exposure to neurotoxic substances.

Population stratification by different African populations could enhance clinical neurological observations in PD. Eastern Africa still has exceptional rural non-sedentary populations such as the Tanzanian Hadzabe, one of the last hunter-gatherer tribes in the world, and the Maasai, semi-nomadic pastoralists. Local Tanzanian medical staff and residents, to the best of our knowledge, have never identified a case of PD in the Hadzabe. A largely similar observation applies to the much more numerous Maasai (numbered at ~1 million). Over 40 years of clinical practice in the area have identified just two cases of PD of Maasai origin, both patients being non-pastoralist and with higher education.unpublished data A common factor in the two tribes is a lack of occupational exposure to insecticides, whereas tribe-specific genetic risk factors are unknown. Although life expectancy is shorter in (semi)nomads, it may also imply a lower-than-average risk of PD in those individuals minimally exposed or unexposed to pesticides. It is anticipated that the frequency of PD in SSA is set to increase because of younger onset of disease, ageing, different risk genes, and toxic exposures particularly pesticides. To better understand this increase, large-scale and long-term studies on genetic predisposition and environmental toxin exposure in PD are needed. Also, inclusion of the world’s vanishing ethnic groups with a nomadic or non-pastoralist lifestyle may still help to increase understanding of what causes PD.

To learn a lesson from a neurological disease with a low frequency in persons of African ancestry living in Africa, multiple sclerosis (MS) is estimated to occur there at a rate of only 1–2/100,000 in contrast to 100/100,000 in high income countries^52^. A recently published study identified Epstein-Barr virus (EBV) infection in late teens or early twenties as an essential risk factor for the development of MS^53^. EBV infection typically occurs in early childhood in Africa^54^ and its causal role with malaria in the pathogenesis of Burkitt’s Lymphoma was among the first to demonstrate a role for viruses in malignancies. It is likely that the same environmental exposure to EBV infection in early childhood in Africa is providing protection against the later risk of developing MS. In a disease of unknown etiology, studies involving “the absence of” may just be as important as studies involving “the presence of”. Consequently, involving ethnic groups in Africa with a relative absence of PD may help increase our understanding of the causes of PD.

### Artur F. S. Schuh, MD, PhD, Brazil

Much has been said about PD as being probably the fastest-growing neurological disorder, which led some authors to call it a pandemic^55^. The most common late-onset sporadic form of the disease is widely recognized as a result of an interaction between genetic and environmental factors, and the only way to change this pandemic is to study these many factors. Fortunately, in recent years, massive efforts have been made to understand the genetic factors driving the disease - efforts with the virtue of including previously underrepresented populations and the potential to point out new pathophysiological pathways and treatments. However, the genetic component may explain only around one-third of the disease risk^26^, and it is implausible that it is responsible for the alarming rising incidence observed in previous decades. Preventing new cases and slowing down this rising curve should be a priority, which can be achieved by also dissecting out environmental factors.

In this regard, there is strong evidence to implicate pesticides as a significant environmental factor associated with the disease, especially paraquat, rotenone, and organochlorines^56–58^. Overall, pesticides may increase the risk of PD and cause an earlier onset and premature death^59–61^. Among them, paraquat is one the most studied and is widely used as a herbicide in many crops worldwide^62^. Acute poisoning can cause death in humans, and chronic exposure has been associated with PD in epidemiology studies^63–66^. Paraquat undergoes redox cycling, producing an excess of oxidative and nitrosative stress, which harms the mitochondria and endoplasmic reticulum and causes apoptosis^62,67^. The molecule is similar to MPP+, the active compound of MPTP, which causes selective damage to substantia nigra^42,68^. Many pathophysiological processes usually associated with PD have been replicated by paraquat models, such as suppression of proteasomal degradation, phosphorylation of parkin, and alpha-synuclein modification and accumulation^62,69^. Interestingly, even developmental exposures seem to produce nigrostriatal toxicity later in life, which is boosted by combination with other pesticides^70^.

Even though the scientific community is aware of its risks, paraquat is still widely used in many countries. Pesticides are loosely regulated in Latin America, where the prevalence of PD seems even higher compared to the Global North and Asia. For example, Brazil is one of the biggest consumers of pesticides in the world, with hundreds of agrochemicals allowed here and forbidden in other countries. Studying populations from developing countries with significant exposure to pesticides, some banned in other countries, represents an excellent opportunity to understand their relationship with PD and to provide insights into disease pathogenesis. Also, efforts should be made to develop reliable biomarkers of chronic and long-term exposure to pesticides, considering it can happen many years before the onset of the disease. Finally, multi-omic population studies, combining genome and exposome (a measure of all the exposures of an individual over their entire lifetime from conception, and how these relate to health), may represent a significant step to disentangling the complex network of interactions leading to PD.

## Toxic alpha-synuclein accumulation and spreading

Over 20 years ago, it was discovered that misfolded alpha-synuclein is the primary constituent of Lewy bodies^71^, and that pathogenic variants in the alpha-synuclein gene (*SNCA*) cause familial PD, thereby linking genetics to PD for the very first time^72^. These discoveries sparked years of further research demonstrating that alpha-synuclein accumulation is not only a hallmark of PD, but that it can also cause neurodegeneration, and forms part of PD’s pathobiology, as commented on below by two contributing authors. The authors comment on the link between alpha-synuclein and mitochondrial dysfunction, the spreading of alpha-synuclein in PD and highlight the utility of imaging tools to detect, better understand and monitor this spreading.

### Nobutaka Hattori, MD, PhD, Japan

Most neurodegenerative diseases such as PD, Alzheimer’s disease and Progressive Supranuclear Palsy manifest protein toxicity as one of their critical pathogenic mechanisms, the details of which remain unclear. By systematically deconstructing the nature of toxic proteins, we aim to elucidate and illuminate some of the essential mechanisms of protein toxicity from which therapeutic insights may be drawn. In PD, alpha-synuclein as a potential prion-mimicking protein has been reported. Αlpha-synuclein can adopt a β-sheet rich structure that forms toxic oligomeric aggregates that accumulate within neurites in the central nervous system and the peripheral nervous system. Such alpha-synuclein can be secreted, taken up by neighboring cells (bodies, dendrites, or axons), and thus induce seeding and spread of the toxic oligomers. Suppose abnormal alpha-synuclein is generated in the intestinal tract and inflammation occurs in the intestinal tract^73^. In that case, it is thought that the seed may become elongated and enter the bloodstream or spread to the brain by ascending the vagus nerve or sympathetic nerves. Many people can eliminate the abnormal alpha-synuclein seed, but if they cannot do so, the seed potential is thought to increase and propagate. In fact, it has been reported that T cells that recognize synuclein peptides are more common in PD, making it highly likely that some immunological mechanism is at work.

Recently, *CHCHD2* as a causative gene for hereditary PD has been identified^74^. *CHCHD2* mutants result in reduced oxygen consumption and ATP production. Although CHCHD2 is localized to mitochondria and is known to be involved in the electron transfer system, brain pathology of individuals with the *CHCHD2* T61I mutation has shown an accumulation of alpha-synuclein throughout the brain. In addition, the co-expression of alpha-synuclein with the CHCHD2 mutant in a Drosophila model resulted in increased alpha-synuclein accumulation and a shortened lifespan. In the knockout mouse model, expansion of alpha-synuclein and p62 has been observed. Therefore, a hypothetical scenario of the two possible series of molecular events is postulated intervening between either synaptic alpha-synuclein deposition leading to induction of mitochondrial dysfunction, or mitochondrial functional deficits leading to accumulation of alpha-synuclein at the synapse^6^. Notably, in both situations, Ca^2+^ rise and production of oxidative stress mediators are pivotally involved in the interconnection between mitochondrial impairment and alpha-synuclein synaptic pathology. Abnormal alpha-synuclein seed and mitochondrial dysfunction are closely related. In other words, the possibility that mitochondrial dysfunction may induce protein toxicity should also be considered.

### Irena Rektorová, MD, PhD, Czech Republic

Based on a recent hypothesis^75^, alpha-synuclein in PD spreads in a prion-like fashion either from the gastrointestinal tract all the way to the brain following the Braak staging (so-called “body first” PD or dementia with Lewy bodies; DLB) with more malignant and symmetric course of the disease, or the brain pathology starts asymmetrically in the amygdala/limbic system and from there it spreads to the substantia nigra, locus coeruleus and other brain (cortical and subcortical) structures and the body via the autonomic nervous system (so-called “brain first” PD) with a presumably less malignant disease course. Our work is focused on studying which of these two options is the most plausible. We do this performing behavioral-magnetic resonance imaging (MRI)-immunohistochemical studies in several models of PD including a transgenic TNWT-61 mouse model overproducing human alpha-synuclein in the brain^76,77^, a toxic methamphetamine model showing massive loss of TH-stained cells in the striatum^78^ or a rotenone model that follows Braak staging with alpha-synuclein spreading from gut to the brain via the vagal dorsal motor nucleus (DMN)^79^.

We used MRI and the diffusion kurtosis imaging (DKI) method which precisely evaluates non-gaussian diffusion of water molecules in gray matter and is an indicator of the heterogeneous environment with restrictions to diffusion. We confirmed that DKI is a sensitive translational marker of alpha-synuclein and alpha-synuclein-induced brain pathology early in the course of the disease (in preclinical stages)^79^ as well as a dynamic marker for monitoring the spread of brain pathology in all of these animal models and in humans with PD of various cognitive subtypes^77,79,80^. Mean kurtosis values correlated with the amount of alpha-synuclein in the thalamus of TNWT-61 mice^76^. However, once alpha-synuclein reached the cortex and cognitive symptoms occurred, neurodegeneration (brain atrophy) was the major player on the scene^78–80^.

Another question still remains to be answered: How does alpha-synuclein *spread in the brain*? It has been hypothesized that brain atrophy progression in Lewy body diseases is shaped by (increased) connectivity and local vulnerability, with alpha-synuclein spreading trans-synaptically, via brain networks^81^. Synaptic plasticity can be studied by functional MRI (fMRI). Resting state fMRI is characterized by low-frequency BOLD (blood oxygen level dependent) fluctuations and various analytical methods can be used for studying large-scale brain networks connectivity. Temporal dynamics of large-scale brain networks alterations can be further assessed by dynamic fMRI^82^ and EEG microstates (MS); the latter representing transient, quasi-stable patterns of EEG that provide us with detailed information about spatial and temporal characteristics of EEG within well-described brain networks^83^. Hyperconnectivity/ hyperactivity of motor and particularly cognitive brain networks have been documented in early stages of PD with normal cognition^84,85^ as well as in prodromal stages of Alzheimer’s disease or frontotemporal dementia^86^ or prodromal DLB^83^. These alterations affect disease-specific vulnerable networks and are considered as compensatory mechanisms, however representing rather maladaptation. In line with this notion, we have demonstrated that increased occurrence of visual network EEG MS in prodromal DLB is associated with lower dominant alpha frequency (which is a supportive diagnostic biomarker of DLB)^83^. This network overactivity seems to decrease as the disease progresses^86,87^.

Taken together, our work indicates that diffusion MRI may detect early microstructural changes that reflect alpha-synuclein-induced brain changes already in preclinical stages of PD and can be used to monitor brain pathology spreading until major neurodegeneration occurs. We hypothesize that this brain pathology spreads via large-scale disease-specific brain networks that show hyperconnectivity (hyperactivity) in early disease stages.

## A complex interaction of multiple factors

To date, the vast majority of PD research has been conducted based on the assumption that PD is a single disease^88^. However, the complex nature of PD has led some, including four contributing authors, to reconsider PD as multiple diseases (instead of a single entity), triggered by complex interactions of many factors that cumulatively push cells past their tipping point.

### Mohamed Salama, MD, PhD, Egypt

PD is best described as a complex disease, of which the complexity is observed on different layers. The first layer of complexity can be seen in its nature; our understanding of PD has changed recently from being a localized neurologic disorder to accepting it as a systemic disease with multiple affections of various systems which reflects an advanced understanding of disease initiation and progression^89^. Another layer of complexity is expressed in the diverse clinical subtypes and even different phenotypes within each of these subtypes^90^. The new appreciation of PD as a mixture of different disorders motivated Farrow and colleagues to conceptualize what they called the “Parkinson’s Diseases Mountain Range model”^88^. It is generally believed that PD develops in response to the interaction of genetic and environmental factors, which has been proven previously in several works including research done by our group in Egypt^91^. The conventional patho-mechanistic model adopted for developing PD is the initial trigger (most probably a genetic risk) that increases the vulnerability to the effect of an environmental factor. This linear–chronological model, however, will not be able to accommodate the recent definition of PD as a diverse and complex mixture of disorders.

I believe that a “threshold model” could better embrace the different mechanisms for developing PD replacing the old (trigger and modifiers) linear one. In this model, the detrimental factor for developing the disease is the threshold after which cells will go into the cycle of degeneration. So, we could have different types of triggers that may lead to the development of the disease. These different triggers—that can work simultaneously—will build up stress on different systems until reaching the damage threshold, which usually does not start in the same time point in all systems, hence, developing the initial stage(s) of the disease. This could justify the earlier non-motor signs of PD that reflect early, non-midbrain damage. The idea of having a threshold, that once exceeded leads to damage, has been widely accepted as a mechanism for neurodegenerative disorders^92^.

We understand now that the disease initiation process is not following a single model. So, in one case, a pathogenic gene variant could interact with different environmental factors causing the disease (gene-environment interaction)^93^. In another case, different genetic variants could interact to cause the disease (gene–gene interaction)^94^, or different pollutants can interact to lead to the disease (environment-environment interaction)^95^. This can be even extended to involve more factors e.g., social stressors, metabolic diseases . etc. This model could allow us to adopt a more holistic – exposomic understanding of factors leading to PD instead of looking for a single interaction, which cannot be validated given the complex exposures a human being could have throughout their lifetime.

Given the diversity in causes, mechanisms and disease processes, it seems mandatory to adopt a more diverse stratification strategy for PD cases. This strategy should accept having different diseases within the spectrum of PD. This better understanding will be reflected in precision medicine approaches tailored to each specific sub-type’s features and needs.

### George D. Mellick, PhD, Australia

The fundamental question regarding the biological basis for PD has led to tensions between geneticists and toxicologists, clinical “lumpers” and “splitters” and pathologists using very strict or more liberal diagnostic criteria. We now need to move beyond disciplinary boundaries to make progress. PD should be considered a syndromic spectrum of many different primary conditions. We know this because rare inheritable forms of Parkinsonism are genetically validated to involve different processes. Genetics has provided many critical clues to etiology since linkage studies in the Contursi kindred heralded the synuclein era^72^. Similarly, the “discovery” that toxins such as MPTP can induce Parkinsonism in humans and the identification of environmental risk factors demonstrate that non-genetic variables also have substantial impact. The truth is that every person living with PD has *a unique expression of the dysfunction and precise balance of factors contributing to this idiosyncratic disease journey*.

Excepting that there are unique combinations of contributing factors, there are also commonalities, which can inform our thinking and help to focus research efforts.

i. PD is progressive and degenerative. Whilst death of dopaminergic neurons is an obvious consequence of the pathology, etiological investigations need to extend well beyond these cell types and brain tissue. The spread of synucleinopathies is important evidence that the initial problem(s) likely arise from areas outside the brain^96,97^.
ii. PD involves the transformation of a “normal” homeostatic process past a tipping point towards a pathological spiral into cellular degeneration; a cascade of secondary and self-reinforcing sequalae such as neuroinflammation, abnormal protein aggregation and metabolic imbalance. Where precisely this is initiated remains elusive, and it may well differ from person to person. The fact that an identical genetic lesion can lead to different pathologies attests to this^98^.
iii. The disease is triggered many years before symptoms of Parkinsonism become obvious and is ongoing in many people who never present with any symptoms^99^.

I believe that appreciating the commonalities while embracing each patient’s idiosyncrasies is key to understanding causal triggers and etiologically informed interventions.

### Christine Klein, MD, Germany

When Bastiaan Bloem invited me to jointly write a Seminar “Parkinson’s Disease” for the Lancet in 2020, we both felt that the more appropriate title for this piece would have been “Parkinson’s Disease*s*”^100^, reflecting not only the plethora of known—mostly genetic—causes of clinical syndromes resembling idiopathic PD but also the fact that each person with PD suffers from his/her own PD not only in terms of disease expression but also cause and modifying factors. Clearly, genetics has been a key driver in our understanding of the etiology of PD and now as a contributor to first attempts at targeted treatment, all of this sparked by the discovery of alpha-synuclein mutations as the first established monogenic cause of PD 25 years ago^72^.

With additional forms of monogenic PD discovered in rapid succession, it quickly became obvious that there are multiple primary causes of PD^101^, which collectively explain almost 15% of PD(s)^102^. and Westenberger et al. in preparation Elegant functional work on shared pathways of the encoded proteins has led to the notion that multiple different pathogenic events converge on one (or more) final common pathway(s) resulting in dopaminergic neurodegeneration and, eventually, the clinical manifestation of PD.

After alpha-synuclein toxicity, mitochondrial dysfunction is probably the next best-established factor in the etiology of PD. Very recently, mitochondria were found to be directly connected to alpha-synuclein conversion from monomeric to oligomeric states in neurons with intracellular seeding events occurring preferentially at mitochondrial membranes where the mitochondrial lipid cardiolipin triggered the oligomerization of mutated alpha-synuclein^103^. However, it has also been demonstrated that “mitochondrial forms” of PD due to pathogenic variants in *PRKN* do not necessarily result in the formation of Lewy bodies, although typical Lewy body pathology has also been described in carriers of biallelic pathogenic *PRKN* variants^104^. To my mind, these findings do not contradict each other but rather support the notion that PD(s) is/are etiologically diverse.

Monogenic forms of PD, at least those that appear to be fully penetrant (such as *PRKN* pathogenic variants or alpha-synuclein triplications), raise the intriguing question as to when PD begins in carriers of such variants. In keeping with the recent biological classification of Huntington’s disease characterizing individuals for research purposes from birth starting at Stage 0 (individuals with a pathologically expanded repeat but without any detectable pathological change)^105^, one may postulate that there are “congenital” forms of PD, as well. This notion has major implications not only for our understanding of (some of) the etiology/ies of PD but also its preclinical phases, offering a much larger and much earlier window of potential intervention. A knock-in Huntington’s disease mouse model (HdhQ7/Q11) showed clear changes in cortical circuit physiology shortly after birth, which was “self-corrected” in the second week of life. Treating pups during the first week of life with an ampakine, thereby correcting the glutamatergic circuit defect, prevented the development of Huntington’s disease-like signs in these mice^106^. These findings suggest that functional abnormalities at the earliest stages of life can potentially be rescued and that treatment of (inherited) neurodegenerative diseases might have to start at the soonest possible time point.

Even with my geneticist’s hat on, I cannot reasonably postulate that “all PD” is genetic even though some of the ‘causal’ PD genes have been shown to play a role also in rare variant burden and even common variant risk^107^. Indeed, we have seen fascinating and independently confirmed developments also in the field of genetic risk factors and polygenic risk scores^26,108,109^ contributing to the etiology of PD. Furthermore, it is conceivable that pathogenic events occur and accumulate over an individual’s lifetime: For example, we have recently demonstrated a relationship between mitochondrial variant burden and development of PD in carriers of heterozygous *PRKN* and *PINK1* pathogenic variants^110^.

Finally, while the field is preoccupied with finding causal and risk factors of PD, I would like to encourage the study and identification of protective and compensatory mechanisms, counteracting the development of PD and, thus, “interfering with” or even serving as a counterpart of its etiology. Some of the protective factors proposed include anti-inflammatory agents, antioxidants, calcium channel agonists, inhibitors of alpha-synuclein aggregation, neurotrophic factors and protective lifestyle factors, such as coffee drinking however this area of research is not yet well developed and requires further study^111^. Recently, “polygenic resilience” has been shown to reduce the penetrance of PD polygenic risk factors with a higher polygenic resilience score being associated with a lower risk for PD^112^. Intriguingly, also the effects of pathogenic variants causing monogenic PD can be mitigated by protective factors. For example, tobacco use and black tea consumption have independent but additive effects on delaying age of onset in carriers of pathogenic *LRRK2* variants^113^.

### A. Jon Stoessl, MD, Canada

More than 30 years ago, my mentor Donald Calne suggested that PD was a syndrome and not a single disease^114^. This has become increasingly obvious if for no other reason than the identification of several monogenic forms of PD, not all of which are associated with alpha-synuclein pathology. That said, and despite the recent failures of monoclonal antibodies targeting misfolded alpha-synuclein^115,116^, there can be little doubt that alpha-synuclein misfolding plays a critical role in a majority of PD, with numerous mechanisms postulated to lead to impaired clearance of misfolded protein. However, it may be worth asking whether PD is a synaptopathy that is associated in most cases with abnormal alpha-synuclein deposition, in others with impaired mitochondrial function, and perhaps in others with unknown underlying pathophysiology.

Some environmental contributors to PD may result in abnormal aggregation of alpha-synuclein (e.g., air pollution^117^; viral infection^118^). Regardless of which mechanism is proposed, hypotheses to date, including that of prion-like propagation of Lewy pathology, have for the most part failed to account for the (partial) neuronal selectivity of PD. Despite the undeniable importance of pathology in non-dopaminergic neurons, one cannot ignore the selective vulnerability of midbrain dopamine neurons and their consistent pattern of involvement, including asymmetry, even in genetic cases. High terminal arborization may contribute^119^, but any theory on cause must take into consideration the evidence that degeneration of dopaminergic neurons likely begins in nerve terminals, not in neuronal cell bodies^35,120^. Therefore, a focus in the future should be on changes that occur at the nigrostriatal terminal and those factors that may contribute not only to the vulnerability of dopamine neurons, but also on the somatotopic selectivity within that population. From that perspective, the role of corticostriatal activity has curiously received inadequate attention and deserves more^121^.

Three other areas deserve further attention in determining why people get PD. One is the role of infection and neuroinflammation. Alpha-Synuclein production and misfolding are triggered by infection^118^ and alpha-synuclein expression is required for Type 1 interferon responses^122^. It is thus of interest that exposure of mice to low levels of SARS-CoV-2 virus renders them susceptible to subtoxic doses of MPTP^123^. Mitochondria, whose dysfunction is the other key proposed culprit, particularly in recessively inherited forms of PD, are required for antigen presentation and that function is repressed by PINK1 and Parkin^124^. A second area that has been largely ignored is the contribution of developmental changes (see ref. ^125^ for a recent review) and stressors that occurred earlier in life (e.g. ref. ^126^) to the later appearance of selective neurodegeneration. In this respect, it is of great interest that in Huntington’s disease, while clinical manifestations do not occur until much later, there is evidence of extensive marked developmental pathology^127^. Finally, theories on PD have been excessively neuron-centric. It would be of great value to explore the contributions of other cells, particularly astrocytes^128^. Astrocytes play a major role in brain metabolism and synaptic function^129^ and are subject to mitochondrial oxidative phosphorylation defects in PD^130^. In addition to alterations in neurotransmitter reuptake and participating in the synaptopathy that may underlie PD, astrocytic dysfunction may contribute to impairment of glymphatic function and additional defects in clearance of misfolded protein. Astrocytes derived from induced pluripotent stem cells in patients with LRRK2 PD express increased alpha-synuclein, resulting in altered calcium homeostasis and increased cytokine release upon inflammatory stimulation^131^ and display altered morphology of extracellular vesicles and altered morphology and distribution of multi-vesicular bodies, with overaccumulation of phosphorylated alpha-synuclein and reduced trophic support and viability of dopamine neurons^132^. Astrocytes from *LRRK2* knock-in mice have abnormal trafficking of glutamate transport^133^ and are impaired in their ability to internalize and degrade fibrillar alpha-synuclein^134^. Prevention of microglial-mediated conversion of astrocytes to the pro-inflammatory state by a GLP1R agonist is protective in both preformed fibril and human alpha-synuclein transgenic mouse models of PD^135^.

## The microbiome–gut–brain axis and possible origin points

### Ai Huey Tan, MD, PhD and Shen-Yang Lim, MD, Malaysia

The heterogeneity of PD continues to be a major conundrum in our field. Differences in the phenotypes at presentation (e.g., the presence/absence of prodromal features such as REM sleep behavior disorder [RBD], constipation and/or hyposmia, and the variability in age of onset and family history) and during the disease course (e.g., rapid vs. slow progression, differential response to dopaminergic medication, presence/absence of non-motor symptoms such as cognitive impairment and autonomic dysfunction which substantially impact clinical outcome) all suggest that there might be differences between individuals with PD, in the “starting point” (i.e., the anatomical location and triggers/causative factors), and in the pattern, speed and extent of spread of PD pathology.

With regards to differences in the starting anatomical location, there has been a growing body of literature on a central (i.e., “brain-first”) vs. peripheral (i.e., “body-first”) origin of PD pathology. The discovery, in the 1980s, of alpha-synuclein deposits in the enteric nervous system (ENS) of PD patients^136^, and subsequent observations that constipation and alpha-synuclein deposits in gastrointestinal biopsies can predate the diagnosis of PD by decades, as well as the marked peripheral autonomic nervous system imaging abnormalities with relative sparing of the nigrostriatal dopaminergic system in patients with prodromal PD (isolated RBD)^137–140^ have implicated the gut as a possible site of origin in PD. It has been postulated that insults acting on the gut could trigger the misfolding and aggregation of alpha-synuclein in the ENS, subsequently propagating into the brain via prion-like cell-to-cell transfer, with the vagus nerve serving as a conduit^141^. Imaging, neuropathological and animal/experimental studies have provided evidence both for and against this hypothesis, while the epidemiological association between full truncal vagotomy and PD risk remains inconclusive^142^. We can perhaps speculate that these mixed findings suggest that a single theory for PD origin is less likely, and central or peripheral degenerative processes may occur at varying timepoints and degrees in different patients. Although earlier autopsy studies by Braak et al. suggested a dual-hit hypothesis (i.e., simultaneous peripheral and central origin with entry points through the dorsal motor nucleus of the vagus [DMNV] and the olfactory bulb [OB], respectively)^143^, a recent re-analysis of post-mortem datasets revealed the lack of concomitant DMNV and OB pathology, suggesting that the pathologic process starts in either location, but rarely simultaneously^144^. Recent studies also indicate that, once begun, dysfunction and pathology can spread bidirectionally, from gut-to-brain, as well as from brain-to-gut^145,146^.

Besides anatomical heterogeneity, inter-individual differences in triggers/causative factors and pathogenic processes are likely to be at play. For example, there is convincing evidence that gastrointestinal inflammation occurs in PD^147–149^, at least in a subset of patients, which can be due to alterations in the composition and/or activities of the gut microbiome, as well as genetic variation (e.g., in *LRRK2*)^142,150,151^. In turn, an inflammatory gut environment is postulated to promote aggregation of alpha-synuclein in the ENS, as well as inducing gut hyperpermeability to toxins and other factors, with far-reaching effects on the brain (e.g., disrupting the blood-brain barrier and triggering a cascade of events culminating in neuroinflammation and neurodegeneration)^142^. Over the past decade, a swathe of studies in PD patients have been published reporting alterations in gut microbiome composition and function^152–154^ with, for example, enrichment of Enterobacteriaceae that express highly immune-stimulatory lipopolysaccharide (that activate Toll-like receptors) or produce curli (an amyloidogenic protein that can template α- synuclein aggregation in the gut)^154,155^; and depletion of bacteria producing beneficial/neuroprotective molecules such as short-chain fatty acids (deficits in which are linked to constipation, gut barrier dysfunction and inflammation)^152–154^. Corroborating these (largely correlative) human observations, animal models suggest that gut microbial-related factors can contribute crucially to PD-like pathogenesis^142,155^. However, definitive proof that they are causative in human PD will likely require large-scale community-based epidemiological studies with multiple sampling over many years to unravel the evolution of gut-related changes in PD. Whether and how lifestyle factors (caffeine intake, cigarette smoking, environmental toxin exposure) that modulate PD risk impact the gut microbiome is also ripe for study^156^.

Importantly, although gut inflammation and alpha-synuclein aggregation might be common events, we believe that PD ensues only when additional contributing factors, such as host genetic vulnerability or ageing, are present^155,157,158^, with ethno-geographic factors also playing a role^153,159^. Ultimately, a deeper understanding of potentially modifiable factors and events operating along the microbiome-gut-brain axis will open up new ways to prevent or change the course of this disabling disease^142,160^.

## A combination of social, biological and environmental factors

### René L. Vidal, PhD, Chile

Movement disorders (MDs) encompass heterogeneous nervous system conditions that cause either an excess of movement or a paucity of voluntary and involuntary movements^161^. The most prevalent MD, PD, affected over 6.1 million people in 2016, and the number of affected individuals have increased 2.4-fold from 1990 to 2016 and continues to grow^162^. In 2017, the economic burden of PD only in the USA was U$51.9 billion^163^. Most MDs feature a long prodromal phase, providing unique opportunities to identify significant risk factors for early detection and intervention, to ultimately improve patient care.

For unknown reasons, Chile, with its partially isolated environments combined with socioeconomic inequalities in rural and urban sectors, has the highest prevalence of PD in Latin America^164^. Ethnically, the Chilean population has an admixed genetic composition of European and South Amerindian ancestries, e.g., Aymaras and Mapuche. These unique ancestral combinations may be involved in the prevalence of MDs. For these reasons it is very relevant to dissect the social, environmental, and genetic factors associated with MDs such as PD in Chile or other South Amerindian populations.

During the past decade, epidemiological and demographic data from Chile, has been used to generate a unique and publicly accessible resource: a nationwide de-identified individual-level electronic health record database. In addition, we have access to clinical statistics (i.e., inpatient services) from the Ministry of Health through the Department of Health Statistics and Information (DHSI) and to environmental factor exposure data (i.e., registry of contaminants by geographic districts) through the Ministry of Environment and others. We have identified a population of more than 37,000 PD cases over the last 20 years who are mainly concentrated in overpopulated or industrialized regions, which once again demonstrates the impact of environmental factors on the development of this type of pathology. Other important epidemiological estimates such as regional disease prevalence, progressions (retrospectively), comorbidities, mortality, social determinants, and disease economic burden also arise as relevant factors involved in PD progression.

Moreover, it is important to consider how biological factors such as genomic variants, gene expression profiles, protein networks, among others associate with the phenotypes observed in individuals with PD. In this line, we recently investigated potential biomolecules in blood samples such as Insulin-like growth factor 2 (IGF2) and autophagic pathways which could play central roles in disease pathogenesis. Our preliminary work has shown a significant decrease in autophagy activity together with a drastic downregulation of IGF2 in peripheral blood mononuclear cell from Chilean persons with PD^165^.

Finally, in PD there are several subcategories related to severities and progressions, and its biological basis is largely unknown. For this reason, it is very important to validate the biological factors in human PD samples and in short-lived disease models at different times of disease progression, which may contribute to understanding the molecular basis associated with differing severity and progression of this disease. I believe that the combinations of biological factors such as genetic variations, protein modifications, autophagic dysfunction, alpha-synuclein accumulation and growth factor levels among others in blood samples, together with environmental and social factors could explain the heterogeneity of motor and nonmotor symptoms observed in PD patients.

## Concluding remarks

**Fig. 1:**
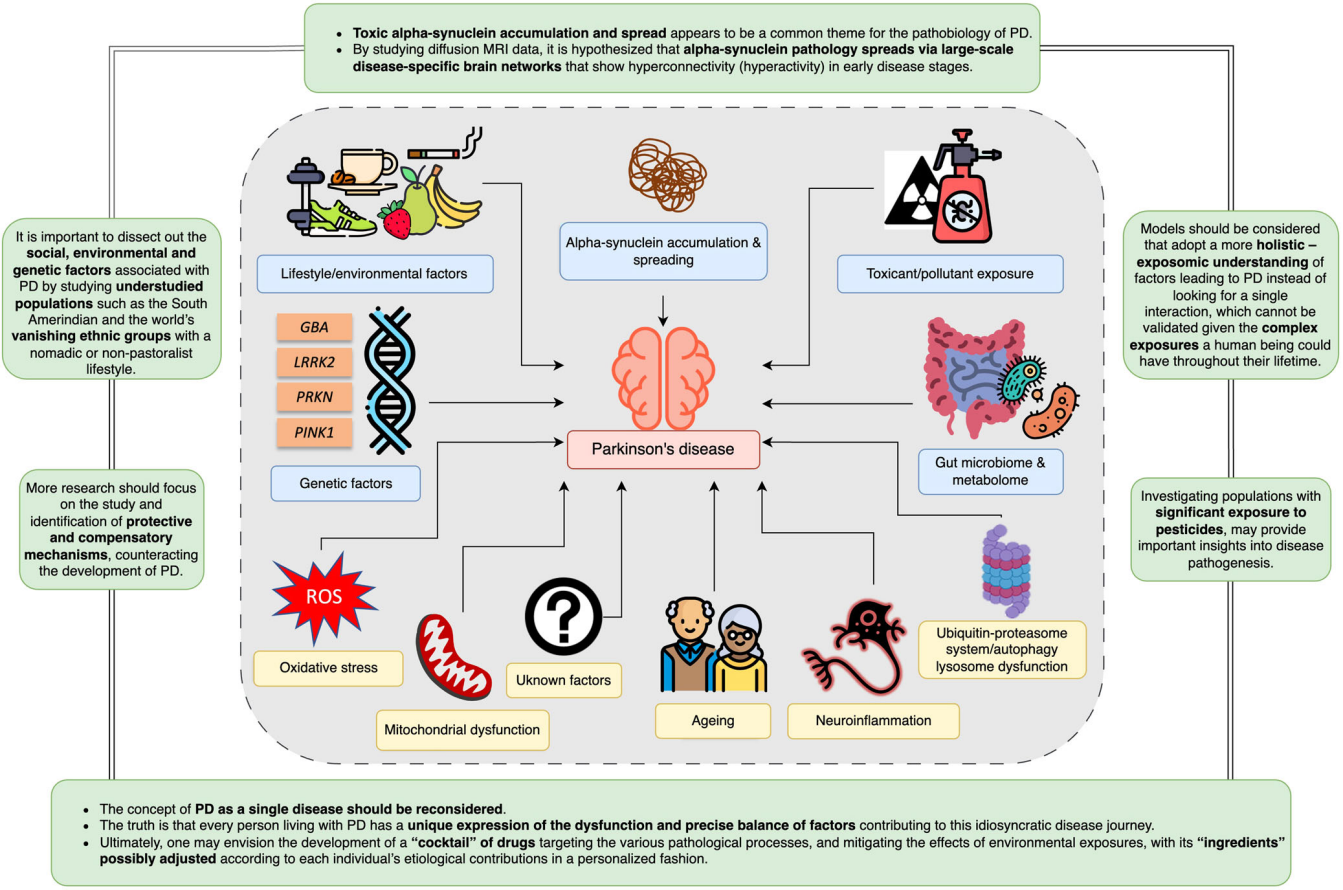
Summary of the main factors, as highlighted in this article, contributing to the complex basis of Parkinson’s disease. The gray box around all these factors represents the notion that multiple or all of these factors may collectively contribute to disease onset. Perspectives, future directions, and take-home messages related to these discussed factors are highlighted in green boxes.

It is clear from the personal viewpoints above that there are a myriad of differing opinions on the biological basis of PD, as illustrated in Fig. 1. This shows that the PD experts are not in agreement with what exactly goes wrong for someone to develop the disorder. While some views overlap in terms of considering genetic factors, alpha-synuclein accumulation, exposure to mitochondrial toxins and neuroinflammation as key etiological drivers, we still do not know what exactly the cause of PD is. It should be noted that most of the views were from researchers with MD, PhD backgrounds which may have introduced a bias in the responses.

We can consider that the etiology of the disorder is multifactorial and due to variances in individual genetic make-up, environment and lifestyle, is different in each person with PD. With the more or less highly penetrant monogenic or strong environmental causes, our etiologic understanding currently represents only the tip of the iceberg. Paradoxically, it will require both a lumper’s as well as a splitter’s approach to fully embrace the etiology of PD. Additional layers of omics studies, including exposomics, are warranted to further explore and potentially disentangle different etiologic contributions and their (epigenetic) changes over an individual’s lifetime. Consequently, this means that each person may have their own unique form of the disease.

From a translational perspective, with a view to more effective treatment or even cure, one may envision the development of a “cocktail” of drugs targeting alpha-synuclein accumulation, restoring GBA levels and mitochondrial function, and mitigating the effects of environmental exposures, with its “ingredients” possibly adjusted according to each individual’s etiological contributions in a personalized fashion.

## Supplementary information

Reporting-summary

## Data Availability

No datasets were generated or analyzed for this article.
